# Une localisation rare du lipome au niveau parotidien: à propos d'un cas

**DOI:** 10.11604/pamj.2018.31.154.14605

**Published:** 2018-10-31

**Authors:** Siham Alaoui Rachidi, Anas Lahlou Mimi, Nizar El Bouardi, Youssef Lamrani Alaoui, Meriem Boubbou, Mustapha Maaroufi, Badr Alami

**Affiliations:** 1 Service de Radiologie, CHU Hassan II, Fès, Maroc

**Keywords:** Lipoma, brain MRI, parotid, Lipome, IRM cérébrale, parotide

## Abstract

La localisation des lipomes au niveau parotidien est très rare. Nous rapportons un nouveau cas avec une revue de littérature concernant un patient de 55 ans, qui a consulté pour une masse au niveau de la région parotidienne évoluant depuis quatre ans. À la palpation nous avons trouvé une formation de consistance molle, mobile et indolore. Le patient a bénéficié par la suite d'une imagerie (échographie et IRM), d'où le diagnostic final d'un lipome parotidien a été retenu ; le traitement était conservateur sur le choix du patient.

## Abstract (en)

Lipomas localization in the parotid region is very rare. We here report a new case of a 55-year old patient, presenting with a mass in the parotid region that had progressed over the past 4 years and a literature review. A soft, mobile and painless mass was detected on palpation. The patient underwent imaging examinations (ultrasound and MRI), that enabled to retain the diagnosis of parotid lipoma. The patient decided to have conservative treatment.

## Introduction

Les lipomes sont des tumeurs bénignes des tissus mous, siégeant le plus souvent au niveau de la partie haute du dos, l'abdomen et aux épaules [1]. Sa localisation au niveau parotidien est rare, représentant moins de 1,5% [2]. Histologiquement, il s'agit d'une prolifération adipocytaire cloisonnée dans une capsule fibreuse. A travers ce cas et revue de littérature, nous rappelons les différents aspects cliniques, diagnostiques et thérapeutiques de cette localisation tumorale rare.

## Patient et observation

**Figure 1:**
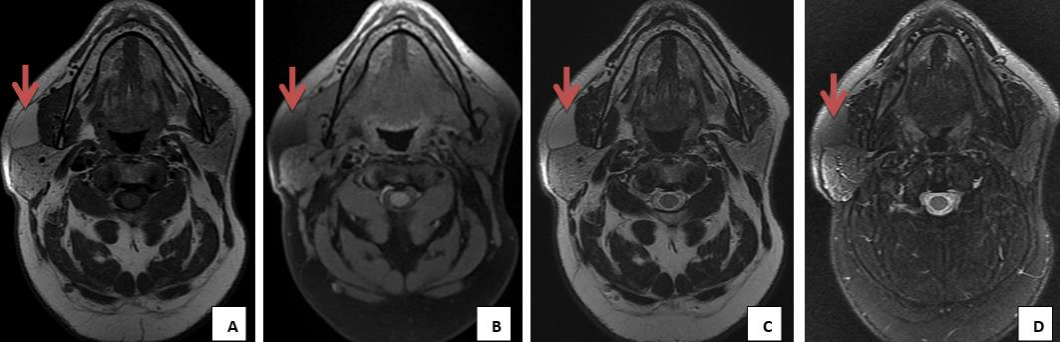
IRM parotidienne en coupe axiale T1 avant (A) et après saturation de graisse (B), et coupe axiale T2 avant (C) et après saturation de graisse, (D) processus tumoral parotidien droit, au dépend de sa portion superficielle, de forme ovalaire, bien limité, de contours réguliers, homogène, décrit en hyper signal T1 et T2, s'effaçant après saturation de graisse

**Figure 2:**
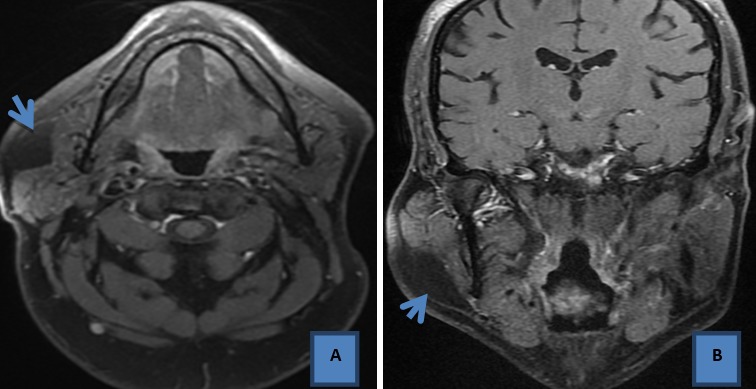
IRM parotidienne du même patient, après injection de gadolinium, en coupe axial T1 (A), coronale T1 (B)

M. Ali. EL.F, âgé de 55 ans, sans antécédents pathologiques notables consultait pour une tuméfaction de la région parotidienne droite évoluant progressivement depuis quatre ans. A l'examen clinique, on a objectivé la présence d'une masse localisée de consistance molle, mobile et indolore mesurant approximativement 2cm de grand axe, avec un signe de Nélaton négatif. Il n'y avait pas d'asymétrie faciale, et le reste de l'examen ORL était sans particularité, notamment pas d'adénopathies cervicales. Une échographie parotidienne réalisée initialement a montré la présence d'une formation échogène hétérogène au dépend de la glande parotidienne droite, non vascularisée au Doppler couleur qui était difficilement caractérisable d'où la nécessité de compléter par une IRM parotidienne. Cette dernière a mis en évidence un processus lésionnel localisé de la portion superficielle de la glande parotidienne droite, mesurant 3cm de grand axe, ovalaire, bien limité, de contours réguliers, homogène, décrit en hyper signal T1 et T2, s'effaçant après saturation de graisse, non rehaussé après contraste. Il n'y avait pas d'adénopathies loco-régionales (Figure 1, Figure 2). Le patient n'a pas accepté une intervention chirurgicale et il a préféré une simple surveillance.

## Discussion

Les lipomes de la région cervico faciale sont rares et surviennent souvent dans le triangle cervical postérieur et la face. Ils sont dans la majorité des cas superficiels, sous-cutanés. Ils représentent moins de 5% de toutes les tumeurs bénignes de la glande parotide [3]. On note une prédominance masculine, avec un sexe ratio de 4/1. Ils surviennent généralement entre 40 et 50 ans [4]. Leur développement s'effectue à partir du tissu graisseux de la glande. Parfois ils refoulent et infiltrent le parenchyme glandulaire. Contrairement à une lipomatose qui correspond à une infiltration graisseuse diffuse de la glande salivaire, généralement bilatérale, symétrique et caractérisée histologiquement par l'absence de capsule fibreuse [5, 6]. Notre cas correspond à une personne de sexe masculin âgée de 55 ans, ce qui rejoint la littérature. Le diagnostic clinique est très difficile à établir car aucun signe clinique ne le distingue des autres tumeurs bénignes de la glande parotide. Généralement ils sont asymptomatiques, on peut avoir une déformation du relief pré-auriculaire. Lorsqu'ils deviennent volumineux, des douleurs fugaces sont parfois décrites [6]. Il faut savoir que seule l'exérèse chirurgicale apportera un diagnostic de certitude [7]. La tomodensitométrie apporte une aide précieuse au diagnostic, en montrant une masse parotidienne homogène, bien encapsulée, de densité négative entre -50 et -100 unités Hounsfield [8]. L'imagerie par résonance magnétique est actuellement l'examen de choix dans l'exploration de la pathologie tumorale des glandes parotides avec une plus grande sensibilité et spécificité comparativement au scanner, elle donne une localisation précise de la tumeur et oriente sur sa nature sans toutefois remplacer un diagnostic histologique que seule la chirurgie apportera [9]. Vu que notre patient n'a pas accepté la chirurgie, on a retenu le diagnostic sur les critères sémiologiques typiques en IRM, sans avoir recours aux données histologiques. Le diagnostic préopératoire peut parfois aider par ponction-biopsie percutanée à l'aiguille fine. Mais, rarement réalisée car elle présente un taux élevé de faux négatifs et souvent l'interprétation des fragments prélevés est délicate [9]. Le traitement de ces lipomes reste chirurgical, ce qui n'est pas toutefois consensuel [1]. Plusieurs techniques sont discutées dans la littérature, entre autre la parotidectomie totale, avec conservation du nerf parotidien, l'énucléation ou la tumorectomie avec une marge de tissu sain [5, 9]. Le taux de récidive des lipomes intra-parotidiens après chirurgie est de l'ordre de 5% [2].

## Conclusion

Les lipomes de la parotide sont rares. L'imagerie par résonance magnétique reste l'examen complémentaire de choix, qui permet grâce à une étude multiplanaire et une résolution spatiale de caractériser une lésion parotidienne et d'apprécier ces rapports. Cependant, le diagnostic de certitude reste toujours histologique.

## Conflits d’intérêts

Les auteurs ne déclarent aucun conflit d’intérêts.
